# Protein Phosphatase-1 Regulates Expression of Neuregulin-1

**DOI:** 10.3390/biology5040049

**Published:** 2016-12-02

**Authors:** Tatiana Ammosova, Kareem Washington, Jamie Rotimi, Namita Kumari, Kahli A. Smith, Xiaomei Niu, Marina Jerebtsova, Sergei Nekhai

**Affiliations:** 1 Center for Sickle Cell Disease, Howard University, Washington, DC 20059, USA; tatiana.ammosova@howard.edu (T.A.); jerotimi@gmail.com (J.R.); namita.kumari@howard.edu (N.K.); kasmith8403@aol.com (K.A.S.); xniu@howard.edu (X.N.); 2 Department of Medicine, Howard University, Washington, DC 20059, USA; 3 Yakut Science Center for Complex Medical Problems, Yakutsk 677019, Russia; 4 Department of Human Genetics, Howard University, Washington, DC 20059, USA; kareem.washington@howard.edu; 5 Department of Microbiology, Howard University, Washington, DC 20059, USA; marina.jerebtsova@howard.edu

**Keywords:** neuregulin-1 transcription, protein phosphatase-1, NIPP1, CDK9

## Abstract

Protein phosphatase 1 (PP1), a cellular serine/threonine phosphatase, is targeted to cellular promoters by its major regulatory subunits, PP1 nuclear targeting subunit, nuclear inhibitor of PP1 (NIPP1) and RepoMan. PP1 is also targeted to RNA polymerase II (RNAPII) by NIPP1 where it can dephosphorylate RNAPII and cycle-dependent kinase 9 (CDK9). Here, we show that treatment of cells with a small molecule activator of PP1 increases the abundance of a neuregulin-1 (NRG-1)-derived peptide. NRG-1 mRNA and protein levels were increased in the cells stably or transiently expressing mutant NIPP1 (mNIPP1) that does not bind PP1, but not in the cells expressing NIPP1. Expression of mNIPP1 also activated the *NRG-1* promoter in an NF-κB-dependent manner. Analysis of extracts from mNIPP1 expressing cells by glycerol gradient centrifugation showed a redistribution of PP1 and CDK9 between large and small molecular weight complexes, and increased CDK9 Thr-186 phosphorylation. This correlated with the increased CDK9 activity. Further, RNAPII co-precipitated with mNIPP1, and phosphorylation of RNAPII C-terminal domain (CTD) Ser-2 residues was greater in cells expressing mNIPP1. In mNIPP1 expressing cells, okadaic acid, a cell-permeable inhibitor of PP1, did not increase Ser-2 CTD phosphorylation inhibited by flavopiridol, in contrast to the NIPP1 expressing cells, suggesting that PP1 was no longer involved in RNAPII dephosphorylation. Finally, media conditioned with mNIPP1 cells induced the proliferation of wild type 84-31 cells, consistent with a role of neuregulin-1 as a growth promoting factor. Our study indicates that deregulation of PP1/NIPP1 holoenzyme activates NRG-1 expression through RNAPII and CDK9 phosphorylation in a NF-κB dependent manner.

## 1. Introduction

Protein phosphatase 1 (PP1) is an abundant cellular serine/threonine phosphatase that regulates multiple cellular processes. Its holoenzyme consists of a catalytic subunit bound to a host of regulatory subunits which determine PP1 localization and activity [1]. Recent genome-wide promoter binding analysis showed that PP1 holoenzyme is targeted to hundreds of cellular promoters by its major nuclear regulatory subunits, PP1 nuclear targeting subunit (PNUTS), nuclear inhibitor of PP1 (NIPP1) and RepoMan, indicating PP1’s potential to regulate the expression of many cellular transcripts [2]. We previously showed that host cell PP1 is involved in the regulation of viral promoters of HIV-1 and Ebola viruses [3,4,5]. Our studies also showed that PP1 can participate in host cell transcription by dephosphorylating the C-terminal domain of RNA Polymerase II (RNAPII) [6]. We also showed that PP1 dephosphorylates and activates cell cycle-dependent kinase 9 (CDK9), a kinase subunit of positive transcription elongation factor-b (P-TEFb) [7]. In addition, we showed that PP1 regulatory subunit NIPP1, targets PP1 to RNAPII [8]. Thus, RNAPII-associated PP1 can potentially regulate host cell transcription by controlling phosphorylation of RNAPII or CDK9. NIPP1 can silence transcription as it constitutes a part of a protein silencing complex that includes the transcriptional repressor, Embryonic Ectoderm Development (EED) protein and Histone Deacetylase 2 (HDAC2) [9,10]. NIPP1 can also activate transcription as it induces expression of mesenchymal genes in HeLa cells [11]. Knockdown of the PP1 catalytic subunit as well as inhibition of PP1 enzymatic activity will interfere with multiple PP1 functions and is likely to be toxic. Therefore, these methods are not generally suitable for the investigation of the role of PP1 in transcription.

We recently developed a panel of PP1-targeting small molecules that activate PP1-dependent HIV-1 transcription [12,13]. Here, we analyzed the cellular proteome of T lymphoblastoid cell line (CEM T cells) treated with a small molecule activator of PP1 (SMAPP1) [13]. We found neuregulin-1 (NRG-1) to be among proteins whose expression was increased by SMAPP1 treatment. NRG-1 is a heparin-binding, neurotrophic factor that regulates both development and life/death of various cells and tissues. NRG-1 binds to and activates the EGF-tyrosine kinase receptor erbB2–erbB3 (Her2–Her3) heterodimer, which belongs to a family of receptors primarily involved in cell proliferation and differentiation and was recently implicated in cardiac dysfunction [14]. The *NRG-1* gene produces at least 15 different NRG-1 isoforms arising from alternative splicing and multiple promoters [15]. While downstream signaling by NRG-1 proteins is well studied, transcriptional regulation of the *NRG-1* gene is poorly understood. Chronic stress, exercise training, estrogen deprivation, vitamin D and antipsychotic drugs increase NRG-1 expression [16,17,18,19]. The only study that has characterized the *NRG-1* promoter showed that it contained Sp1 and NF-κB binding sites and presumed to be activated by Sp1 and NF-κB factors [20]. An HIV-1 promoter that contains Sp1 and NF-κB binding sites, is upregulated by PP1 [5,21]. Therefore we asked whether PP1 and its regulatory subunit NIPP1 are involved in the activation of *NRG-1* transcription. Both transient and stable expression of mutant NIPP1 (mNIPP1) that does not bind PP1, induced mRNA and protein expression of *NRG-1*. Using an *NRG-1* promoter-controlled reporter, we found that mNIPP1 expression turned on the reporter in an NF-κB-dependent manner. Analysis of the extracts, obtained from mNIPP1 expressing cells and separated on glycerol gradients, showed that PP1 and CDK9 co-localized to the same molecular weight fraction where, additionally, CDK9 was phosphorylated on Thr-186. Cellular activity of CDK9 was elevated in cells stably expressing mNIPP1. Taken together, our study demonstrated that PP1 and its regulatory subunit NIPP1 controlled transcription of *NRG-1* gene. Targeting PP1 with non-competitive small molecule inhibitors represents a useful way to modulate NRG-1 expression.

## 2. Materials and Methods

### 2.1. Materials

Antibodies to NRG-1 (1:1000 dilution for Western blot (WB)) were from Abcam (cat #ab80237, Cambridge, MA, USA). Antibodies to α-tubulin (1:5000 dilution for WB) were from Sigma-Aldrich (Atlanta, GA, USA). Rabbit anti-PP1 antibodies (1:500 dilution for WB) were from Calbiochem (Gibbstown, NJ, USA). Antibodies to CDK9 (1:2000 dilution for WB) were from Santa Cruz Biotechnology (Santa Cruz, CA, USA). Phospho-Thr-186-specific anti-CDK9 antibodies (1:1000 dilution for WB) were a gift from Dr. Qiang Zhou (University of California, Berkeley, CA, USA). Expression vectors for Enhanced Green Fluorescent Protein (EGFP)-fused central domain of NIPP1 (NIPP1-(143-224)) or mNIPP1 (NIPP1-(143–224) V201AF203A (RATA mutant)) were described [22]. mNIPP1 mutated within its RVxF motif has a low binding affinity for PP1 [22]. Anti-NIPP1 antibodies were a gift from Dr. Monique Beullens (University of Leuven, Leuven, Belgium) and were described in [23]. The expression vectors containing NRG-1 promoter (PBS 769) and the NF-κB mutated NRG-1 promoter (PBS 769 NF-κB mut) followed by *Lac Z* reporter [20] were a gift from Dr. Thomas Schmitt-John (University of Aarhus, Aarhus, Denmark). All salts and other reagents were from Sigma-Aldrich.

### 2.2. Sample Preparation for Mass Spectrometry Analysis

CEM T cells were collected and lysed with whole cell lysis buffer (50 mM Tris-HCl, 500 mM NaCl, 1% NP40, 0.1% sodium dodecyl sulphate(SDS)) supplemented with protease inhibitors (Sigma-Aldrich). The insoluble nuclear material was removed by centrifugation at 21,000× *g* for 20 min. The supernatant was collected and protein concentration was measured using the bicinchoninic acid (BCA) protein assay (Thermo Fisher Scientific, Rockville, MD, USA). Proteins were precipitated with cold acetone. The samples were centrifuged at 13,000× *g* for 10 min. Pellets were dried for 10 min at room temperature and then re-suspended in 100 mM ammonium bicarbonate buffer containing 10 mM dithiotreitol (DTT). The samples were heated at 95 °C for 5 min to be reduced, then alkylated with 15 mM iodoacetamide and digested with trypsin gold (Thermo Fisher) overnight at 37 °C. Discovery Supelco (Sigma-Aldrich) C18 column was used for peptides separation. Si-propylsulfonic acid (SCX) resin (POROS 50 HS, Perspective Biosystems) column was prepared in a pipette tip and was equilibrated with 0.5% formic acid in 0.25% acetonitrile (equilibration buffer). Samples were eluted by varying concentrations of NaCl solution and dried in a Speed-Vac concentrator centrifuge (Thermo Fisher Scientific).

### 2.3. Mass Spectrometry and Data Analysis

The mass spectra of the peptides were obtained with a data-dependent 4-event scan method (a survey Fourie Transfrom (FT)-Mass Spectrometry (MS) parent scans followed by FT-MS/MS tandem mass spectrometry). Protein identifications was carried out using Proteome Discoverer 1.2 software in combination with the SEQUEST protein database search engine and International Protein Index (IPI) Human Protein Database (version 1.79). A sequential database search was performed using the human FASTA database. Only peptides with a cross-correlation (XCorr) cutoff of 2.6 for [M + 2H]^2+^, 3.0 for [M + 3H]^3+^ and a higher charge state were considered. These SEQUEST criteria typically result in a 1%–2% false discovery rate (FDR). The FDR was determined by searching on a decoy database. We used SIEVE 2.1 software (Thermo Fisher, Waltham, MA, USA) for label-free quantitative analysis of the high resolution MS spectra produced by Orbitrap MS scans. We then explored protein networks in SMAPP1-treated cells using results from SIEVE 2.1 in Ingenuity Pathway Analysis (Qiagen, Valencia, CA, USA). Venn diagrams were constructed using Genevenn freeware (http://genevenn.sourceforge.net/).

### 2.4. Transient Transfections of Cell Cultures

The 84-31 cells derived from HEK293 cells with stable expression of mNIPP1 (mutant EGFP-fused NIPP1 K193-197A/V201A/F203A/Y335D) have been described previously [24]. HEK 293T, 84-31 and 84-31-mNIPP1 cells were maintained in Dulbecco’s Modified Eagle Medium (DMEM) containing 10% fetal bovine serum (FBS) and antibiotics (all from Invitrogen). CEM T cells were maintained in RPMI-40 media with FBS and antibiotics. Transfections were performed using a Ca^2+^-phosphate protocol with the indicated reporter plasmids [21]. Transfected cells were cultured for an additional 48 h before harvesting.

### 2.5. Quantitative RT-PCR

RNA was isolated from cells by using a TRizol reagent (Invitrogen Life Technologies, Carlsbad, CA, USA). Reverse transcription (RT) was performed using the Superscript™ RT-PCR kit (Invitrogen Life Technologies). Random hexamers were used for RT reaction. Primers for PCR of individual target sequences were designed for NIPP1 (forward: AGAATTCAACACTGCCACA; reverse: CACCCGCTTCTTCTTGACTG), NRG-1 forward: TGGGAATGAATTGAATCGAAA; reverse: TGGCAGAGGCACTGTCATT), and β-actin forward: GCGGGAAATCGTGCGTGCGTGACATT; reverse: GATGGAGTTGAAGGTAGTTTCGTG). The −769 to −89 portion of Nrg1 type 1 promoter 769 bp upstream off the translation initiation codon was used. It has two NF-κB binding sites and multiple Sp1 sites [20]. Primers were generated to amplify a product no larger than 200 nucleotides, incorporating two different exons, and spanning no less than 0.5 kilobases of intron nucleotides. For real-time PCR, the SYBR^®^ Green Super Mix (Bio-Rad Laboratories, Hercules, CA, USA) was used in PCR reactions as instructed by manufacturer. Amplification of target cDNA was performed in an amplification format of melting at 95 °C for 15 s, annealing at 55 °C for 30 s, and extension at 72 °C for 45 s for 45 cycles on a MyiQ™ real-time PCR detection system (Bio-Rad Laboratories). The obtained Ct numbers were converted to the cDNA copy numbers using as calibration amplification of NIPP1-expressing plasmid and primers for NIPP1.

### 2.6. Centrifugation in Glycerol Gradients

The 84-31 and 84-31 mNIPP1 cells were grown on 100 mm plates, then lysed with 0.5 mL of whole cell lysis buffer (50 mM Tris-HCl, pH 7.5, 0.5 M NaCl, 1% NP-40, 0.1% SDS) supplemented with a cocktail of protease inhibitors (Sigma-Aldrich). Cell lysates were clarified by centrifugation for 30 min at 10,000× *g* and loaded on top of 10% to 30% glycerol gradient. The glycerol gradient buffer contained 20 mM HEPES-KOH, pH 7.9, 150 mM KCl, 0.2 mM EDTA. The gradients were spun in a Sorvall XL-90 Ultracentrifuge (Beckman Coulter, Brea, CA, USA) with a SW41 Ti rotor at 38,000 rpm for 18 h. Twenty fractions were collected through a needle inserted to the bottom of the tube using fraction recovery system (Beckman Coulter). Proteins were precipitated with 70% trichloroacetic acid (TCA), resolved in 10% SDS-PAGE and analyzed by immunoblotting as indicated.

### 2.7. β-Galactosidase Activity Assay

The β-galactosidase activity was analyzed using a quantitative *O*-nitrophenyl-β-d-galactopyranoside (ONPG)-based assay [21]. Transfection levels were normalized using EGFP. Briefly, cells were washed with PBS and lysed in lysis buffer (20 mM HEPES, pH 7.9, 5 mM EDTA, 0.1% NP-40) for 20 min at room temperature. For β-galactosidase assays, cells were washed with phosphate-buffered saline (PBS) and lysed for 20 min at room temperature in 50 μL of lysis buffer, containing 20 mM HEPES at pH 7.9, 0.1% NP-40 and 5 mM EDTA. Subsequently, 100 μL of ONPG solution (72 mM Na_2_PO_4_ at pH 7.5, 1 mg/mL ONPG, 12 mM MgCl_2_, 180 mM 2-mercaptoethanol) was incubated at room temperature to develop a yellow color. The reaction was stopped by the addition of 100 μL of 1 M Na_2_CO_3_. The 96-well plate was analyzed in a micro plate reader at 414 nm (Lab Systems Multiscan MS).

### 2.8. CDK9 Kinase Activity Assay

CDK9 was immunoprecipitated (IP) from 84-31 and 84-31-mNIPP1 cells with either anti-CDK9 polyclonal antibodies or with pre-immune serum. A portion of the IP product was resolved on 10% SDS-PAGE and immunoblotted with anti-CDK9 antibodies. The remaining IP product was supplemented with (^32^P) ATP (Perkin–Elmer, Waltham, MA, USA) and incubated with recombinant Glutathion S- transferase(GST)-CTD. Products of kinase reactions were resolved on 10% SDS-PAGE and visualized utilizing a Phospho Imager (Perkin Elmer, Waltham, MA, USA).

### 2.9. Immunoprecipitation of RNAPII

The 84-31 or 84-31 mNIPP1 cells were lysed in whole cell lysis buffer (50 mM Tris HCl, pH 7.5, 1% NP-40, 0.1% SDS, 0.25 M NaCl). The whole cell extract (50 mg) was supplemented with 2 μg of anti-RNAPII-CTD monoclonal antibodies recognizing non-phosphorylated CTDo form (clone 8WG16, Sigma-Aldrich) coupled to protein-A/G agarose in Tris-NaCl-NP40 (TNN) buffer (50 mM Tris, pH 8.0, 150 mM NaCl, 0.5% NP-40). Immunoprecipitation was carried out for 2 h at 4 °C. Antigenic complement coupled beads were washed three times with 0.5 mL of TNN buffer and once with 50 mM Tris-HCl, (pH 8.0). Proteins were resolved on 4%–12% SDS-PAGE and transferred to polyvinylidene fluoride (PVDF) membrane for immunoblotting analysis.

### 2.10. Analysis of RNAPII Phosphorylation in Cultured Cells

The 84-31 cells and 84-31 cells expressing mutant NIPP1 were cultured in 24-well plates with Dulbecco’s modified Eagle’s medium (DMEM, Life Technologies) containing 10% (v/v) fetal bovine serum. At 75% confluency, cell cultures were treated with okadaic acid (OA) (0.01 mM or 1 mM), 25 μM flavopiridol or a combination of OA and flavopiridol, for 30 min. After treatment, cells were washed in PBS and lysed with SDS-loading buffer. Lysates were adjusted to equal protein concentration and resolved on 4% SDS-PAGE, transferred to PVDF membrane and analyzed by immunoblotting with 8WG16 (1:1000 dilution), or clone H5 (1:500 dilution) (Abcam) monoclonal antibodies against non-phosphorylated or Ser-2 phosphorylated RNAPII CTD.

### 2.11. Cell Growth Assays

The 84-31 and 84-31-mNIPP1 cells were grown to confluence in a 25 cm^2^ culture flask. The media from the 84-31-mNIPP1 cell culture was removed and spun at 3500 rpm for 15 min. The 84-31 cells were washed with PBS, detached by trypsinization and seeded in a 96-well plate with 2-fold decrement starting from 80,000 cells per well. Where indicated, the dilutions were made with media conditioned with 84-31-mNIPP1 cells. Cells were grown at 37 °C for one to four days, supplemented with 0.5 mg/mL MTT for 2 h. MTT (Sigma-Aldrich) was dissolved in DMSO and the absorbance was measured at 620 nm.

### 2.12. Statistical Analysis

Results are expressed as mean ± SD. Differences between any two groups were compared with the unpaired two-tailed Student’s *t*-test on GraphPad Prism 4.01 software (GraphPad Software, La Jolla, CA, USA).

## 3. Results

### 3.1. NRG-1 Expression Is Induced in Cells Treated with a Small Molecule Activator of PP1 (SMAPP1)

**Figure 1:**
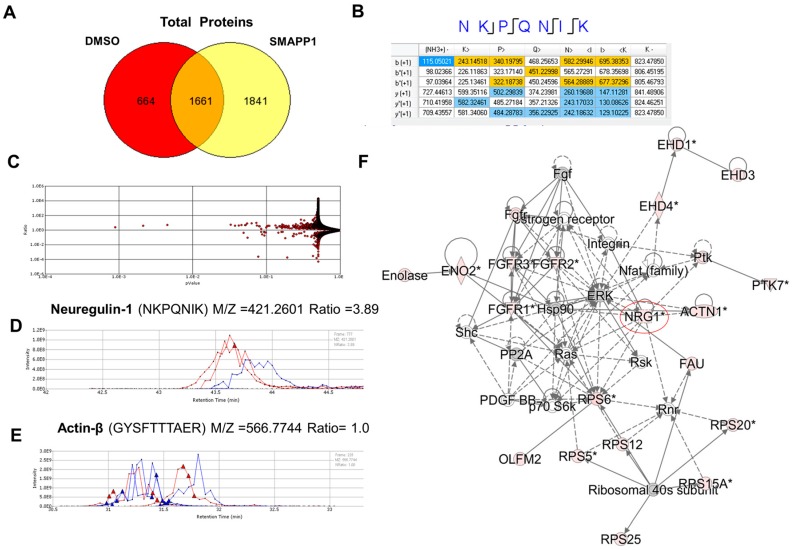
Small molecule activator of PP1 (SMAPP1) induces NRG-1 expression. (**A**) Venn diagram of proteins that were detected in CEM T cells treated with 10 μM SMAPP1 or DMSO as control. Cellular proteins were extracted, reduced, alkylated and trypsinized. Tryptic peptides were separated by ion exchange chromatography and analyzed by high resolution mass spectrometry. Proteins were identified by Proteome Discoverer 1.2. Peptides eluted in 50 mM NaCl are shown on the diagram; (**B**) Tandem mass spectrometry (MS/MS) analysis of NRG-1 derived peptide in Proteome Discoverer 1.2. **Blue** color indicates high confidence and **yellow**—low confidence ions; (**C**) Peptides detected in the 50 mM salt fraction were quantified using SIEVE 2.1. The volcano plot shows ratios of peptides present in the 50 mM salt fraction in SMAPP1 versus DMSO treated samples with corresponding *p*-values; (**D**,**E**) Quantitative analysis of NRG-1 and β-actin expression using SIEVE 2.1. Ion elution profiles are shown in **blue** for control samples and in **red** for the CEM T cells treated with SMAPP1. Panel D shows the elution of the NRG-1-derived ion. Panel E shows the elution of the actin-β-derived ion. Results from two independent experiments are shown. Triangles indicate the time points at which MS/MS was conducted. Integration of the peaks was performed by SIEVE 2.1 and the ratios of the ion peaks in SMAPP1 versus control cells are shown, (**F**) Peptides detected in the 50 mM salt fraction and analyzed by SIEVE 2.1 were exported to Ingenuity software for the analysis of protein networks. A network with NRG-1 is shown. Up-regulated genes are colored in pink.

We recently reported a small molecule activator of PP1 (SMAPP1) that induced PP1 activity in vitro and activated latent HIV-1 provirus in cultured and primary T cells [13]. To gain further insight into the mechanism of SMAPP1, we analyzed proteins expressed in SMAPP1-treated T cells using liquid chromatography followed by FT-MS/MS tandem mass spectrometry [13]. We performed short-gun proteomics on whole cell extracts of SMAPP1-treated CEM T cells trypsinized and fractioned off-line on a cation exchange column. Tryptic peptides were eluted stepwise with sequential column washes in an elution buffer containing no salt, 25 mM, 50 mM, 100 mM, 250 mM and 500 mM NaCl, as previously described [25]. Over 1800 proteins induced by SMAPP1 were present in the 50 mM NaCl fraction of the tryptic hydrolysate obtained from SMAPP1-treated cells (Figure 1A). Among these proteins, we found a peptide corresponding to NRG-1 (Figure 1B). To determine whether this NRG-1-derived peptide was present at higher levels in SMAPP1-treated cells, we quantified it in a relative amount using a label-free approach. For this purpose, we used SIEVE 2.1 software which allows the extraction of selected ions and their quantification by integrating their ion elution profiles (Figure 1C–E). The peptide ion corresponding to NRG-1 (M/Z = 421.2601) was present at approximately a 4-fold higher level in SMAPP1-treated cells as determined by two separate elution profiles (Figure 1D, red color, Ratio = 3.89). The peptide ion corresponding to β-actin (M/Z = 566.774) was present in equal proportion between untreated and SMAPP1-treated lysates (Figure 1E, Ratio = 1.0). We used Ingenuity Pathway Analysis (IPA) software to identify the network of proteins associated with the induction of NRG-1 (Figure 1F). About 60% of the proteins in the network associated with NRG-1 were upregulated in cells treated with SMAPP1 (Figure 1F). Together, these observations suggest that NRG-1 is induced in cells in which PP1 activity is modulated.

### 3.2. Overexpression of mNIPP1 Induces NRG-1 mRNA Expression

**Figure 2:**
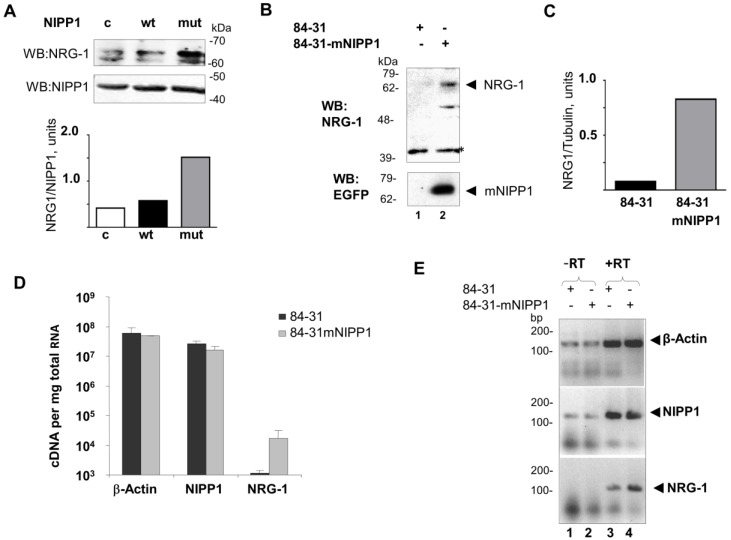
Expression of mutant NIPP1 (mNIPP1) induces NRG-1 expression. (**A**) Transient expression of mNIPP1 increases the protein level of NRG-1. HEK293T cells were transfected with either NIPP1-EGFP or mNIPP1-EGFP expression vectors. Lysates were collected 48 hours after transfection, resolved on 7.5% SDS-PAGE gel and analyzed by Western blot using anti-NRG-1 and anti- EGFP antibodies. Lower panel shows quantification; (**B**,**C**) Stable expression of mNIPP1 increases the protein level of NRG-1. Lysates from 84-31 cells and 84-31-mNIPP1 cells were resolved on 7.5% SDS-PAGE and analyzed by Western blot with anti-NRG-1 and anti-EGFP antibodies. An asterisk indicates the position of the non-specific protein band serving as a loading control. Panel C shows quantification of the results from panel B; (**D**) Quantitative RT-PCR (qPCR) analysis of NRG-1 expression. Total RNA was isolated from 84-31 cells and from 84-31-mNIPP1 cells, reverse transcribed and amplified with primers for β-actin, NIPP1 and NRG-1 as described in Experimental Procedures using SYBR green. Ct values obtained were recalculated to the number of cDNA copies per mg of total RNA. Mean and SD are shown; (**E**) Analysis of the quantitative RT-PCR products on agarose gel. PCR amplified cDNA was resolved on 2% agarose gel and visualized with ethidium bromide staining. Lanes 1 and 2, non-transcribed RNA; and lanes 3 and 4, reverse-transcribed RNA from 84-31 and 84-31 mNIPP1 cells amplified with primers for β-actin, NIPP1 or NRG-1 as indicated.

To determine whether PP1 was involved in the upregulation of NRG-1 at protein and mRNA levels, we transiently expressed NIPP1 or mNIPP1 that has mutations in the central and *C*-terminal domains that rendered it incapable of binding PP1 [22]. Immunoblot analysis showed increased expression of NRG-1 in 84-31 cells transiently expressing mNIPP1 compared to cells expressing NIPP1 or non-transfected cells (Figure 2A compares lane 3 to lanes 1 and 2). Transient expression of NIPP1 and mNIPP1 did not change the expression of endogenous NIPP1 (Figure 2A). We previously generated a stable cell line expressing mNIPP1 by infecting 84-31 cells with the adeno-associated virus expressing mutant NIPP1-EGFP protein and by selecting cells that expressed mNIPP1-EGFP in the nucleus (84-31-mNIPP1 cells) [8]. Expression of mNIPP1-EGFP fusion protein in 84-31-mNIPP1 cells was detected by Western blot (Figure 2B, lane 2).

Stable expression of mNIPP1 has upregulated the expression of NRG-1 (Figure 2B,C). Increased NRG-1 mRNA expression was also detected by real-time RT-PCR in 84-31-mNIPP1 cells whereas expression of β-actin or NIPP1 was not changed (Figure 2D,E). Analysis of the RT-PCR products on 2% agarose gel showed specific amplification of NRG-1 gene product in contrast to equal expression of β-actin or NIPP1 controls (Figure 2E, compare lane 4 to lane 3). Together, these results suggest that expression of mNIPP1 significantly induces mRNA and protein levels of NRG-1.

### 3.3. Transcription from Neuregulin-1 Promoter Was Induced by Expression of mNIPP1

**Figure 3:**
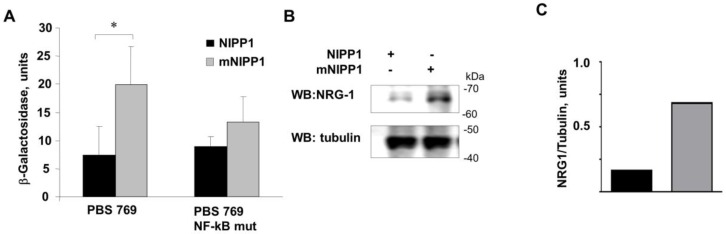
Expression of mNIPP1 increases transcription from the NRG-1 promoter. (**A**) HEK293T cells were transiently transfected with NIPP1-EGFP or mNIPP1-EGFP expressing vectors and co-transfected either with an expression vector in which LacZ expression was controlled by the NRG-1 promoter (PBS 769) or the NF-κB-mutated PBS 769 expression vector. Expression of LacZ was measured and normalized to EGFP expression. Mean and SD are shown. An asterisk (*) shows *p* value ≤ 0.05; (**B**) Expression of mNIPP1 induced endogenous NRG-1 expression in HEK293T cells. Cells were transfected with NIPP1-EGFP or mNIPP1-EGFP expression vectors. Cell lysates were resolved on 10% SDS-PAGE and analyzed by Western blot with anti-NRG-1 and anti-tubulin antibodies; (**C**) Quantification of panel B. NRG-1 expression is shown normalized to tubulin.

To determine whether mNIPP1 expression has an effect on the NRG-1 promoter, we utilized previously described constructs that contain a portion of the NRG-1 promoter (−769 nt to −89 nt) followed by the *LacZ* reporter gene (PBS 769) [20]. Co-expression of PBS 769 with NIPP1-EGFP or mNIPP1-EGFP in HEK 293T cells showed increased LacZ expression in 293T cells co-transfected with mNIPP1-expressing plasmid but not NIPP1 (Figure 3A). In contrast, the NRG-1 reporter with mutated NF-κB sites showed no significant upregulation of LacZ with either co-expression of NIPP1-EGFP or mNIPP1-EGFP (Figure 3A). This suggests that the Sp1-sites are not affected by mNIPP1 expression. To confirm that transient mNIPP1 expression induces NRG-1 protein expression in HEK 293T cells, the cells were transfected with a vector expressing mNIPP1-EGFP or a control EGFP expression vector. NRG-1 expression was analyzed by immunoblotting. Expression of mNIPP1 significantly increased the expression of endogenous NRG-1 (Figure 3B,C) confirming that transient mNIPP1 expression in HEK 293T cells led to increased NRG-1 protein expression. Taken together, these results indicate that mNIPP1 induces transcription from the NRG-1 promoter in an NF-κB-dependent manner.

### 3.4. Expression of Mutant NIPP1 Redistributed PP1 and Increased Thr-186 Phosphorylation of CDK9

**Figure 4:**
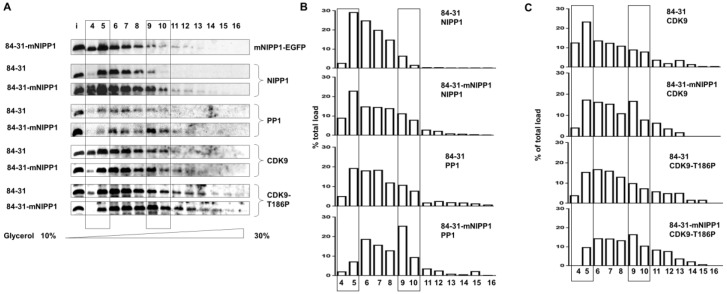
Stable expression of mNIPP1 induces CDK9 phosphorylation and changes in CDK9 and PP1 distribution. (**A**) Western blot analysis of extracts fractionated on glycerol gradients. Whole cell extracts from 84-31 and 84-31-mNIPP1 cells were separated by ultra-centrifugation on glycerol gradient (10%–30%). Fractions were collected, precipitated and resolved on 10% SDS-PAGE, and further analyzed by Western blot with antibodies against EGFP, NIPP1, PP1, CDK9 and CDK9 phosphorylated on Thr-186. Direction of the glycerol gradient is indicated; (**B**) Quantification of NIPP1 and PP1 migration from panel A. Protein expression is shown normalized to total protein load; (**C**) Quantification of CDK9 and CDK9 phosphorylated on Thr-186 migration from panel A. Protein expression is shown normalized to the total protein load.

Transcription of most cellular genes are regulated by P-TEFb [26] which exists as a small or large molecular weight complex. The lower molecular weight kinase active form of P-TEFb consists of CDK9 and cyclin T1, whereas the high molecular weight form of P-TEFb is a protein-RNA complex that includes 7SK RNA, HEXIM1, LARP7 and MEPCE proteins (reviewed in [27]). We analyzed whether mNIPP1 expression has an effect on P-TEFb distribution or CDK9 phosphorylation on Thr-186. Whole cell extracts from 84-31 and 84-31-mNIPP1 cells were separated by ultracentrifugation in glycerol gradient (10%–30%). Fractions were collected and analyzed by immunoblotting (Figure 4). We lysed cells in a buffer with high salt to ensure extraction of the nuclear proteins; some large complexes might have been disrupted. In our previous study, we observed 7SK RNA to migrate in glycerol gradient fractions 12 and higher [4]. Here, the majority of CDK9 migrated in lower molecular weight fractions 4–10, indicating that the large complex of CDK9/cyclin T1 and 7SK RNA was disrupted (Figure 4A). Expression of mNIPP1-EGFP was detected with anti-GFP antibodies (Figure 4, upper panel). mNIPP1 was detected in the lower molecular weight fractions (Figure 4, fraction #4). 

The ratio of expressed mNIPP1-EGFP to endogenous NIPP1 was 1:1 according to Western blot results. We could only detect mNIPP1-EGFP using anti-GFP antibodies. Therefore, the ratio was approximated based on expression levels from anti-NIPP1 rabbit antibodies compared with the expression given by anti-GFP rabbit antibodies. In the extract containing mNIPP1, PP1 was shifted from lower molecular weight fractions 4 and 5 to the higher molecular weight fractions 9 and 10 (Figure 4, PP1 panel). The distribution of CDK9 did not change much (Figure 4, CDK9 panels). There was, however, an increase in Thr-186 phosphorylated CDK9 in higher molecular weight fractions 9 and 10 in 84-31-mNIPP1 cells (Figure 4, CDK9 T186P panel). Autophosphorylation of CDK9 Thr-186 residue activates the transcriptional activity of the enzyme. Association of CDK9 with different complexes is regulated by CDK9 phosphorylation on Thr-186 which is, in part, controlled by PP1 [27]. Overall, these results indicate that the expression of mNIPP1 increased the level of PP1 in the high molecular weight complexes; mNIPP1 also increased the higher molecular weight CDK9-containing complex in which CDK9 was phosphorylated on Thr-186. Thus, both PP1 and Thr-186 phosphorylated CDK9 shifted to larger molecular weight complexes in 84-31-mNIPP1 cells in comparison to wild type 84-31 cells.

### 3.5. Expression of Mutant NIPP1 Induces Cellular Activity of CDK9

**Figure 5:**
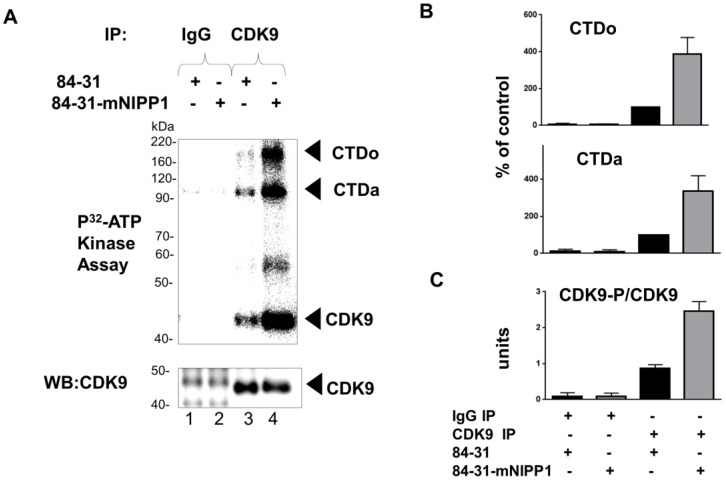
Stable expression of mNIPP1 increases enzymatic activity of CDK9. (**A**) Analysis of CDK9 activity and phosphorylation. CDK9 was immunoprecipitated from 84-31 or 84-31-mNIPP1 cells with either anti-CDK9 polyclonal antibodies (lanes 3 and 4) or with pre-immune serum (lanes 1 and 2). A portion of the immunoprecipitation product (IP) was resolved on 10% SDS-PAGE and immunoblotted with anti-CDK9 antibodies (WB). The remaining IP was supplemented with (^32^P) γ-ATP and incubated with GST-CTD. Reactions products were resolved on 10% SDS-PAGE and visualized using PhosphorImager Storm 860 (Molecular Dynamics). The positions of the CDK9, hypo- (CTDa) and hyper- (CTDo) phosphorylated *C*-terminal domain of RNAPII are indicated by arrowheads; (**B**,**C**) Quantification of phosphorylated CTDo and CTDa bands and the CDK9 phosphorylated band from panel A. Phosphorylated CDK9 was normalized to non-phosphorylated CDK9.

CDK9 activity is regulated by the phosphorylation of multiple serine/threonine residues and its association with the inhibitory large molecular weight complex containing 7SK RNA and HEXIM1 protein [27]. Because CDK9 Thr-186 phosphorylation is required for its enzymatic activity [28], we analyzed the effect of the expression of mNIPP1 on the global enzymatic activity of CDK9 in mNIPP1-expressing cells. CDK9 was immunoprecipitated from 84-31 or 84-31-mNIPP1 cellular extracts and its autophosphorylation and kinase activity towards the GST fusion *C*-terminal domain of RNAPII (GST-CTD) were analyzed. We found that CDK9 precipitated from 84-31 mNIPP1 cells has increased CTD phosphorylation activity and also showed higher autophosphorylation compared to CDK9 precipitated from 84-31 cells (Figure 5, lane 4). Thus, expression of mNIPP1 induced enzymatic activity of CDK9. 

### 3.6. Mutant NIPP1 Associates with RNAPII

**Figure 6:**
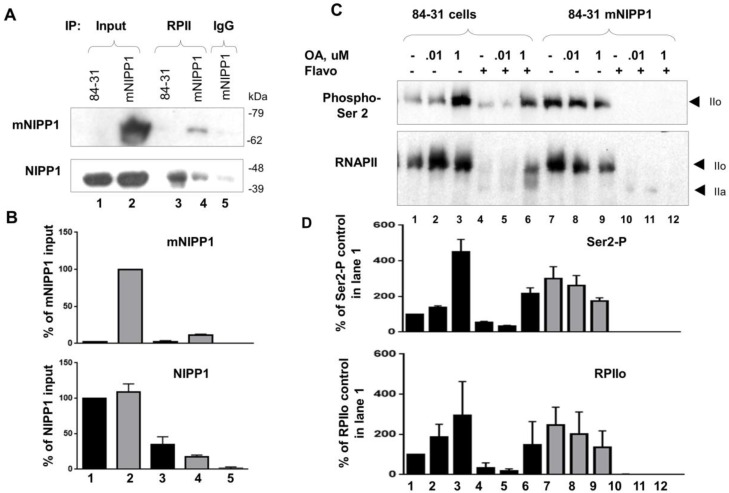
Stably expressed mNIPP1 associates with RNAPII and increases its phosphorylation. (**A**,**B**) mNIPP1 co-precipitates with RNAPII. RNAPII was immunoprecipitated from 84-21 cell extract with 8WG16 monoclonal antibodies. Immunoprecipitated proteins were resolved by 7.5% SDS-PAGE and immunoblotted with anti-NIPP1 antibodies. Panel B shows quantification of the panel A; (**C**,**D**) Expression of mNIPP1 alters the phosphorylation of RNAPII CTD. 84-31 cells or 84-31-mNIPP1 cells were treated where indicated with 25 μM flavopiridol for 30 min to promote RNAPII dephosphorylation. The cell were also treated for 30 min with 0.1 μM or 1 μM okadaic acid (OA) prior to the treatment with flavopiridol, to block PP2A or PP1 phosphatases. Whole cell lysates were prepared, resolved on 5% SDS-PAGE and analyzed by Western blot with Ser-2 phospho-epitope specific antibodies (upper panel) or RNAPII-specific antibodies (lower panel). The hyperphosphorylated (IIo) and hypophosphorylated (IIa) forms of RNAPII are indicated. Panel D shows quantification of panel C.

We previously showed that NIPP1 was associated with RNAPII [8]. Therefore, we analyzed the co-precipitation of mNIPP1 with RNAPII. RNAPII was precipitated with monoclonal antibodies and probed with anti-NIPP1 antibodies. Both NIPP1 and mNIPP1 were detected (Figure 6A,B), suggesting that PP1 targeting to RNAPII can be disrupted by mNIPP1 overexpression and affect RNAPII phosphorylation. To confirm our previous observation that NIPP1 affects RNAPII phosphorylation [8], we analyzed whether mNIPP1 expression affects RNAPII phosphorylation in a PP1-dependent manner. Analysis of RNAPII CTD phosphorylation was carried out in 84-31 cells and 84-31 mNIPP1 cells that were treated with okadaic acid (OA). OA inhibits PP1 at a higher concentration and PP2A at a lower concentration [29]. We also used flavopiridol, a CDK9 inhibitor [30], or a combination of OA and flavopiridol (Figure 6C,D). Treatment of 84-31 cells with 1 μM but not 0.1 μM OA induced RNAPII Ser-2 phosphorylation (Figure 6C,D, lanes 2 and 3). This suggested that inhibition of PP1 induced RNAPII phosphorylation (Figure 6C,D, lane 3). In contrast, RNAPII was highly phosphorylated on Ser-2 in untreated 84-31 mNIPP1 cells or 84-31 mNIPP1 cells treated with 0.1 μM OA (Figure 6C,D, lanes 7 and 8), suggesting upregulated RNAPII phosphorylation in the absence of PP1 inhibition. A brief treatment with 25 μM flavopiridol inhibited RNAPII phosphorylation in both 84-31 and 84-31 mNIPP1 cells (Figure 6C,D, lanes 4 and 10). Addition of 1 μM but not 0.1 μM OA restored RNAPII phosphorylation in 84-31 cells (Figure 6C,D, lanes 5 and 6) but not in 84-31 mNIPP1 cells (Figure 6C,D, lanes 11 and 12), suggesting that PP1 no longer influenced RNAPII phosphorylation in mNIPP1-expressing cells.

### 3.7. Induction of 84-31 Cell Growth in Media Conditioned with 84-31-mNIPP1 Cells

**Figure 7:**
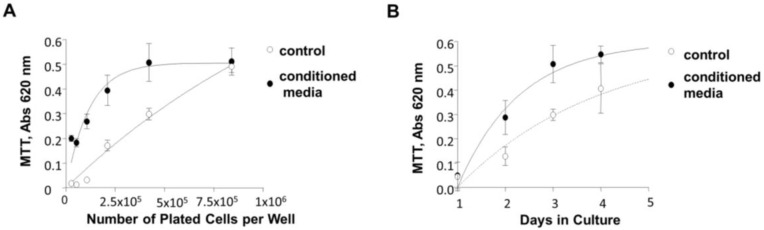
Cell culture medium conditioned with 84-31 mNIPP1 cells increases growth of 84-31 cells. (**A**) Media collected from 84-31-mNIPP1 cells were added to replace complete DMEM media from 84-31 cells. A different starting number of cells showed different rates of growth for 84-31 cells. To monitor cell proliferation, an MTT assay (Sigma-Aldrich) was used; (**B**). Plated 84-31 cells were grown for 4 days and compared with 84-31 cells treated with conditioned medium collected from 84-31-mNIPP1 cells. Cell proliferation was measured at 620 nm using the MTT assay kit. Assays were conducted in 96-well plates and done in triplicates. Cell number and vitality were determined by trypan blue staining using Cellometer (Nexcelom Bioscience, Lawrence, MA, USA). Means ± SD are shown (*n* = 4).

NRG-1 is a neurotrophic factor that induces proliferation of neuronal and epithelial cells [31,32] and, therefore, it may also affect the growth of 84-31 cells which are derived from epithelial HEK293 cells. We investigated whether conditioned media collected from 84-31-mNIPP1 cell culture stimulated the proliferation of 84-31 cells. Although 84-31-mNIPP1 cells grew only 1.2 times faster than the original 84-31 cells (results not shown) [33], 84-31 cells grew greatly (two to four times), when 84-31 cells were incubated in the medium conditioned with 84-31-mNIPP1 cells (Figure 7A,B). This observation indicates that the 84-31-mNIPP1 cells secrete growth stimulating factor(s) that may include neuregulin-1.

Collectively, our results demonstrate that manipulation of PP1 either by treatment with a PP1-targeting small molecule, SMAPP1, or through the expression of mNIPP1, induced NRG-1 expression. Expression of mNIPP1, in turn, increased RNAPII Ser-2 phosphorylation and CDK9 Thr-186 phosphorylation and induced the cellular activity of CDK9.

## 4. Discussion

In the present study, we showed that deregulation of PP1 by SMAPP1 or the expression of mNIPP1 induces the transcription of NRG-1. The over expression of mNIPP1 may have a global effect on gene transcription. We focused, however, on NRG-1 as an example to validate the role of PP1 in the regulation of eukaryotic cell gene expression. Our choice of NRG-1 as a candidate protein was based on its important role in normal development as well as predisposition to diseases. The *NRG-1* gene is located on human chromosome 8 and is approximately 1.4 mega bases long [15]. It produces at least 15 different isoforms due to alternative splicing and multiple promoters. NRG-1 proteins play an essential role in the development of the nervous system, heart and breast [15]. *NRG-1* has been identified as a schizophrenia susceptibility gene [34], and a risk factor for breast cancer [35] and cardiac hypertrophy [36]. Despite the fact that transcription of NRG-1 is complex and tightly regulated, the mechanisms regulating *NRG-1* transcription are poorly understood.

We demonstrated here that *NRG-1* transcription is regulated by PP1. Treatment of CEM T cells with SMAPP1 led to the increase in the abundance of NKPQNIK peptide which matched to the intracellular domain of NRG-1. Although the NKPQNIK peptide is not fully cleaved, its determination was carried by high resolution MS/MS analysis which was conducted in FT mode with accuracy of 0.01 Da that significantly improved the reliability of FT-MS/MS data. We also validated NRG-1 expression at the mRNA level by real-time PCR and protein level by Western blot. Hence, we feel confident that NRG-1 expression was elevated under the condition of PP1 deregulation. NRG-1 was therefore a good candidate for our study. mNIPP1 expression is not likely to increase PP1 activity independently: mNIPP1 might compete with NIPP1 either in promoter-binding or in RNAPII-activating complexes and subsequently displace and re-distribute PP1 between different complexes. For example, it was previously demonstrated that histone deacetylase inhibitor, trichostatin A (TSA) induced activation of the human luteinizing hormone receptor (LHR) gene by dissociating PP1 associated with Sp1 from the LHR promoter in MCF-7 cells [37]. Our analysis of protein complexes in the glycerol gradient showed an increase of PP1 in the higher molecular weight fractions 9 and 10 which correlated with increased CDK9 Thr-186 phosphorylation. The activity of CDK9 is regulated by transient phosphorylation/de-phosphorylation of several serine/threonine residues and by association of CDK9 with different protein-RNA complexes [12]. The cellular activity of CDK9/cyclinT1 is inhibited by 7SK RNA [38,39], and HEXIM1 protein [40,41]. Phosphorylation of CDK9 on Thr-186 residue promotes the binding of 7SK RNA and HEXIM1 [28,42]. This phosphorylation is also required for the enzymatic activity of CDK9 [43]. Transient de-phosphorylation of Thr-186 is required for CDK9 dissociation from the large inhibitory complex and formation of the small complex that activates transcription [44]. CDK9 recruited to RNAPII is phosphorylated on Thr-186 by CDK7 which restores the enzymatic activity of CDK9 and reactivates paused RNAPII [45]. In this study, we observed the re-distribution of CDK9, increased phosphorylation of Thr-186 and increased enzymatic activity of CDK9 in the cells with stable expression of mNIPP1. Although we did not repeat glycerol gradient analysis of 84-31 cells and m84-31 cells, we are confident that re-distribution of active CDK9 increases Thr-186 phosphorylation. Separation by glycerol gradient centrifugation is a good tool in detecting the re-distribution of different proteins when combined with complementing methods and useful in analyzing the components of protein complexes or their activity.

It is possible that NIPP1 is part of a protein complex that specifically silences the *NRG-1* gene. NIPP1 interacts with EED, a transcriptional repressor that binds to the same domain of NIPP1 as PP1, but the binding of EED and PP1 are not mutually exclusive [9]. EED interacts with histone deacetylase (HDAC2), which is also present in a complex with NIPP1 [9]. EED interacts in the context of Polycomb repressive complexes 2 and 3 (PRC2/3) with DNA methyltransferases to promote their binding to the gene targeted for repression [10]. mNIPP1 may preferentially bind EED over PP1 and target it to RNAPII. This, however, cannot explain the induction of NRG-1 transcription by the overexpression of mNIPP1. Alternatively, it is more plausible that PP1 bound to NIPP1 plays a yet unrecognized role in the silencing of the *NRG-1* gene—possibly by controlling the phosphorylation and activity of CDK9, in a similar manner to the Small *C*-terminal domain Phosphatase 1 (SCP1) function in silencing neuronal genes outside the neuronal tissue [46].

While we were not able to detect released NRG-1, we observed increased growth of 84-31 cells in medium conditioned with 84-31 mNIPP1 cells. Soluble NRG-1 was shown to bind to EGF-tyrosine kinase receptors erbB2–erbB3 (Her2–Her3) heterodimer, a family of receptors primarily involved in cell proliferation and differentiation [47,48]. NRG-1 induces growth of neuronal cells [32] and also lung epithelial cells [31]. Thus, it is likely to be released by 84-31 mNIPP1 cells.

Taken together, PP1-NIPP1 complex may be potentially involved in down regulating *NRG-1* transcription through the inhibition of CDK9 activity. Re-distribution of PP1 activates *NRG-1* transcription by increasing the activity of CDK9 in the P-TEFb complex that may function synergistically with NF-κB on the *NRG-1* promoter. We cannot rule out the possibility that PP1 is also involved in silencing the *NRG-1* promoter through binding with other transcriptional factors in a cell-specific manner. The primers for RT-PCR recognized all fifteen isoforms of NRG-1, thus we could not determine whether PP1 is involved in the regulation of all or only a few protein isoforms. We also did not study multiple *NRG-1* promoters, except the promoter region from −769 to −89 which contained NF-κB site. Future study of the different NRG-1 isoforms, more detail investigation of its promoter region and study of proteins associated with PP1, NIPP1, CDK9 and *NRG-1* promoter will allow better understanding of the regulation of NRG-1 transcription. To the best of our knowledge, this is the first report to demonstrate a role for PP1 and its regulatory subunit NIPP1 in the activation of NRG-1 transcription.

## 5. Conclusions

Here, we showed that NRG-1 transcription is upregulated in the cells treated with SMAPP1 or expressing mNIPP1. We observed increased abundance of NRG-1-derived peptide in the SMAPP1-treated cells. Expression of mNIPP1 increased NRG-1 mRNA and protein levels, and also activated the *NRG-1* promoter in an NF-κB-dependent manner. In mNIPP1 expressing cells, PP1 was shifted to larger molecular weight complex and CDK9 Thr-186 phosphorylation was increased. This correlated with the increased CDK9 activity which was detected for immunoprecipitated CDK9 that was probed with GST-CTD substrate. Expression of mNIPP1 also increased RNAPII CTD Ser-2 phosphorylation in the cells. Moreover, RNAPII CTD Ser-2 phosphorylation inhibited by flavopiridol could not be rescued by okadaic acid in mNIPP1 cells. These results suggest that PP1 was no longer involved in RNAPII dephosphorylation in mNIPP1 cells likely due to the PP1 exclusion by mNIPP1 associated with RNAPII. Finally, we found that mNIPP1 cells produced a growth factor that accelerate the proliferation of parental 84-31 cells. This finding was consistent with a role of neuregulin-1 as a growth promoting factor. Taken together, our study point to the role of PP1-NIPP1 holoenzyme in suppression of NRG-1 transcription. Deregulation of this holoenzyme by mNIPP1 expression activates NRG-1 expression through RNAPII and CDK9 phosphorylation in a NF-κB dependent manner. Our findings point to a novel pathway of NRG-1 suppression by PP1. Our study also highlights PP1-targeting small molecules that might be of interest for neurological, proliferative and cardiac disorders centered around NRG-1.
